# Influence of CNT Filler in Polymer Matrix on Impact Damage Propagation in the Volume of Carbon Fiber Laminates

**DOI:** 10.3390/polym17070891

**Published:** 2025-03-26

**Authors:** Egor Morokov, Pavel Shershak, Mikhail Burkov, Alexander Eremin, Elizaveta Popkova, Nikolay Yakovlev, Irina Zhiltsova

**Affiliations:** 1 Emanuel Institute of Biochemical Physics, Russian Academy of Sciences, 119334 Moscow, Russia; iraida_n@mail.ru; 2 Federal State Unitary Enterprise, All-Russian Scientific Research Institute of Aviation Materials, National Research Center “Kurchatov Institute”, 105005 Moscow, Russia; tweek@list.ru (P.S.); lab30@viam.ru (E.P.); nick_62@mail.ru (N.Y.); 3 Institute of Strength Physics and Materials Science of Siberian Branch of Russian Academy of Sciences, 634055 Tomsk, Russia

**Keywords:** polymer composite, CFRP, matrix modification, impact damage, ultrasound, volume imaging, CNT

## Abstract

The addition of nano-sized fillers into the polymer matrix of carbon fiber laminates is considered today as one of the ways of increasing the strength and resistance of the material to mechanical loads. The paper considers the effect of the addition of single-walled carbon nanotubes (CNTs) on the development of impact damage in laminates. Studies of the volume microstructure and its damage were carried out using high-resolution ultrasound imaging. The effect of damage propagation in laminates with an increase in the concentration of CNTs from 0 to 0.5 wt% was shown. The addition of CNTs decreased the area of damage in the upper and lower part of laminates but increased the area of damage in the middle plies. The results were discussed in combination with data on impact histories of laminates.

## 1. Introduction

Carbon fiber reinforced polymer (CFRP) composites are a promising class of materials with outstanding properties such as strength, stiffness, and lightness. These materials find a wide application in various fields, including aerospace, sports equipment, automotive, and others [1,2]. However, CFRP laminate is prone to delamination under mechanical loads due to the low interlaminar fracture toughness. This is primarily determined by properties of the matrix and the fiber–polymer bond. One of the current challenges in the field of composites is to improve interaction between the polymer matrix and reinforcing fibers, increase the fracture toughness, and ultimately improve the crack resistance.

In recent decades, promising studies have been conducted to improve fracture toughness by modifying the polymer matrix [3,4,5], modifying the surface of reinforcing fibers [6,7,8], or by vertically crosslinking (pinned) carbon layers [9,10]. Matrix modification was carried out by incorporating additional nanoparticles into the polymer matrix, namely: carbon multi-walled carbon nanotubes (MWCNT) [3,11,12,13,14], graphene oxide (GO) [15], graphene nanoplatelets (GNP) [16,17], nanoscale clay (NC) [18], short aramid fibers [19,20], and various combinations of them [21,22]. The works showed that the addition of these nanoparticles led to improvements in interlaminar strength. Nanophase evokes the concept of multiscale reinforcement, which connects the boundary layers vertically, leading to the redistribution and dissipation of energy during destruction. Ou et al. [13] demonstrated that the crack front propagated alternately above and below the nanotube-reinforced interlayer. This significantly improves the fracture toughness of the laminates. The mechanism is highly dependent on how the nanotubes were integrated into the matrix. They suggested [13] that directly depositing CNTs on plies as low-density thin layers resulted in significant improvements in interlaminar properties, while mixing the MWCNT with the matrix leads to agglomeration and defects. Liu et al. [21] incorporated graphene oxide and MWCNTs into composite laminates, achieving significant improvement in interlaminar fracture toughness. Koirala et al. [23] used ultra-thin aligned CNT sheets (100 nm) to improve the interlaminarity without changing the weight or thickness of laminates. These studies confirm the positive effect of CNTs on interlaminary fracture toughness. Tehrani et al. [24] showed that the incorporation of MWCNTs into CFRP increased the impact energy absorption by up to 21%. Bedsole et al. [11] showed that the introduction of nanotubes resulted in improvements in fracture toughness under both quasi-static (+34%) and dynamic (+16%) loading conditions. Alshehri et al. [3] found that the lowest delamination initiation force was observed in impact tests for laminates with a pure polymer matrix, and it increased with increasing concentrations of MWCNTs at 0.2 and 0.4 wt% in the polymer. Despite the direct relationship between the concentration of nanofillers in a matrix and their fracture toughness, there is a limit on nanophase concentration. A high affinity of MWCNT can lead to the agglomeration of nanoparticles, reducing the effect of introducing them. In addition, when a filler is introduced, the viscosity of resin increases, affecting the diffusion and impregnation of prepreg during manufacturing. To reduce particle agglomerate, chemical functionalization of nanofillers was used, significantly affecting the dispersion of nanofiller in the matrix [25]. Another possible solution was layer-by-layer application using a spray [21].

Interlayer fracture toughness is especially important for low-velocity impacts, when the main energy of mechanical action is concentrated in the interlayer areas. Under such loads, so-called barely visible damages are formed in the CFRP volume. These damages are not visible from the surface, but they can occupy a large area in the composite volume and significantly reduce its load-bearing capacity. The study of the microstructure of CFRP under low-velocity impact has been challenging since most damage appears in the volume of optically opaque laminate. Therefore, the application of non-destructive techniques for characterizing CFRP looks attractive. Today, quantitative characterization and visualization of impact damage in CFRP can be performed using X-ray [26,27,28,29] and ultrasound [3,30,31,32,33] techniques or thermography [34,35]. The non-destructive approaches solve two problems: the validation of numerical models for predicting impact damage [36,37,38,39,40,41,42], and the monitoring of irreversible changes to the microstructure without cutting and polishing composite samples. Direct experimental results from the non-destructive observation of structural disturbances significantly improve current and future numerical models of CFRP failure prediction. Therefore, in this work, the high-resolution ultrasound visualization technique is used to observe the damage in CFRP reinforced with SWCNTs under low-velocity impact. The features of damage appearance and location in composites with different CNT concentrations are revealed and discussed.

## 2. Materials and Methods

### 2.1. Specimen Preparation

Composites were prepared using biaxial fabric CBX300 (Mitsubishi Pyrofil TR50S 12K, Sacramento, CA, USA) and epoxy binder R&G Epoxy L (GL2 hardener) with the addition of single-walled carbon nanotubes (SWCNT) TUBALL (OcSiAl, Luxembourg), supplied by the manufacturer in the form of a pre-dispersed composition of CNT with ethoxylated alcohol. SWCNTs had a mean diameter of 1.6 ± 0.4 nm and a length of >5 μm.

This CNT batch was added to the epoxy resin and thoroughly mixed before the hardener was introduced. The batch was mechanically mixed with epoxy for 10–20 min and a simple visual homogeneity test was applied, both proposed by the manufacturer. Carbon fabrics were laid out using manual layup (stacking sequence [+45/−45/0/90]_5S_) followed by vacuum bagging and pressing in a thermal press Gotech 7014 under a pressure of 0.7 MPa and a temperature of 80 °C. After a dwell time of one hour, the laminate was removed from the mold, the release film and breather fabric were removed, and post-curing for 24 h at a temperature of 80 °C was performed in a heating chamber. After the post-curing, the specimens for testing were cut on a CNC milling machine Purelogic RM0813 (Voronezh, Russia) with polycrystalline diamond mill with water cooling. One unmodified epoxy laminate and four laminates modified with 0.1, 0.2, 0.3, and 0.5 wt% of SWCNT were manufactured for testing. The specimen geometry of 100 × 150 mm was based on the ASTM D7136 standard [43]. The thickness of laminates was 4.8 mm.

### 2.2. Scanning Electron Microscopy

The microstructure was evaluated using a Quanta 200 3D scanning electron microscope (FEI Company, Hillsboro, OR, USA) (SEM). Preliminary samples of 4 × 4 × 4.8 mm were cut from the laminates and heated in a NETZSCH STA409PC/PG (Selb, Germany) thermogravimetric analysis (TGA) unit at the following conditions: heating from ambient temperature to 873 K at a rate of 15 K/min with an Argon flow rate of 50 mL/min. TGA procedure leads to the decomposition and evaporation of the epoxy matrix while leaving the carbon fibers and CNTs undamaged. Subsequent SEM analysis of the TGA residue allows to visualize CNT distribution.

### 2.3. Mechanical Tests

Low-velocity impact tests were carried out using the Drop Weight Tester HIT230F from Zwick Roell (Zwick GmbH Co.KG, Ennepetal, Germany). The diameter of the impactor was 25.4 mm (1 in). The impact energy was ~30 J with a mass of 5.6 kg.

### 2.4. High-Resolution Ultrasound Imaging

The scanning impulse acoustic microscope SIAM-2011 [30,44] developed and produced by the Institute of Biochemical Physics, Russian Academy of Science (Moscow, Russia), has been applied in experiment. An acoustic lens with a frequency of 50 MHz and angle aperture of 11° provides a lateral resolution of 60 μm in the CFRP volume at the depth of a few mm. Short probe pulses of 25 ns in duration provide an axial resolution of 40 µm. Ultrasound devices can visualize extended cracks and delaminations with a nanoscale gap. The reflection of these defects is complete, making it possible to detect and visualize the damage in a laminated volume. The volume microstructure of laminates can be represented as a set of B-scans and C-scans taken at different depths inside the specimen, which depict the microstructure in vertical and horizontal sections of the laminate, respectively. A detailed description of visualization modes, principles of formation, and interpretation of carbon fiber elements are presented in the work [45].

## 3. Results

The results of the research are the comparison of the impact history values (loads, energies) and data of high-resolution ultrasound visualization of damage in the volume of laminates. A direct comparison of the values of impact loads and energies with the sizes of damage and their localization over the laminate thickness will allow a better understanding of the mechanism of the influence of CNT on damage propagation.

### 3.1. SEM Investigations

**Figure 1:**
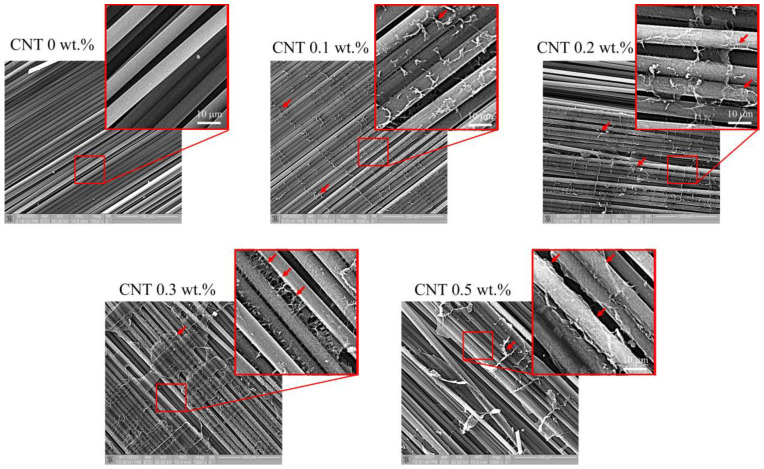
SEM micrographs of laminates with various concentrations of CNT. The CNT conglomerates formed a continuous network linking carbon fibers in the transverse direction. The size of CNT meshes increased with increasing of concentration. Arrows are the CNT conglomerates.

Figure 1 shows SEM micrographs of CNT distribution in laminates. The CNTs were located between carbon fibers and formed cross-links between them. The distribution of CNTs was irregular, with particles congregating and forming a continuous transversal network of group CNTs throughout the laminates. The network increased with increasing filler concentration. Large conglomerates of CNTs surrounded several carbon fibers at concentrations of 0.3 wt% and 0.5 wt%.

### 3.2. Impact Behavior

**Figure 2:**
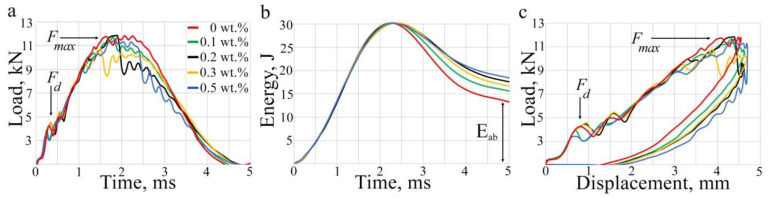
Mechanical test results. Impact force histories (**a**), impactor energy history (**b**), and curves of force versus impactor displacement (**c**). The threshold load of delamination *F_d_* has minimal variability and is equal to 4 kH (**a**). The peak load *F_d_* occurred at the indenter displacement of 0.6 ÷ 0.9 mm (**c**), which is associated with the appearance of the first damages. *F_max_* is the peak force that occurred during the impact; the values of *F_max_* were about 11 kH. The total impact energy was 30 J; however, only part of it was absorbed by the laminates. The absorbed energy *E_ab_* (**b**) increased with increasing CNT concentration.

The force-time histories obtained from the impacts on the laminates are shown in Figure 2. The force-time dependencies of all the impact tests were analyzed in order to evaluate the delamination threshold load *F_d_*, and peak force *F_max_* (Table 1). All the specimens had approximately equal values for the threshold load *F_d_* of ~4 kN and the peak force *F_max_* of ~11.5 kN. The lowest value of *F_max_* of 10.5 kN occurred in laminates containing 0.3 wt% of CNT. It is clear that the higher initial energy *E*_ini_ at force of damage initiation *F*_d_ will result in the lower energy for damage propagation and the lower damages in the laminates. The laminate with 0.3 wt% of CNTs had the higher value of the energy *E*_ini_; however, the peak force *F*_max_ was slightly lower than other specimens. Despite the similar force histories (Figure 2a), the curves of the laminates with 0.2 wt% and 0.3 wt% of CNTs showed a significant drop of load immediately after the peak forces. At the same time, the total displacement of the impactor was about 4.6 mm (Figure 2c). It can be seen that there was an elastic energy component in the energy-time plot (Figure 2b) and that the absorbed energy *E_ab_* increased with increasing of CNT concentration (Table 1); the rebound of the impactor also occurred, as indicated by the curves of force versus impactor displacement (Figure 2c).

### 3.3. Projected Damaged Areas of the Laminates

**Figure 3:**
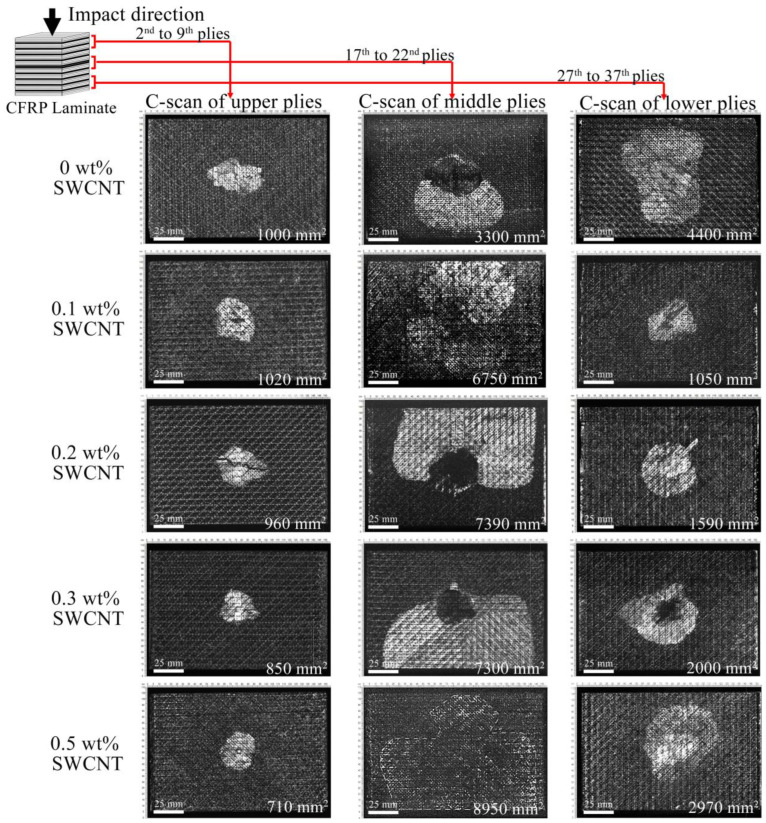
C-scans of the impact damage in the volume of the laminates with various concentrations of CNTs. The images show three zones in the thickness of the laminates with the calculated area of damage. Bright areas in the C-scans correspond to interlayer delaminations. The size of damages in the upper plies (left column) decreased with increasing of CNT concentration. Projected damaged areas in the middle zone (central column) of the laminates increased with increasing of CNT concentration. The laminate with a CNT content of 0.1 wt% has minimal damage in the lower plies (right column); the damage area increases with increasing concentration. The maximum size of delaminations (4400 mm^2^) was observed in the pure laminate.

Impact damage zones in the volume were assessed by visualizing the microstructure over the thickness of laminates. Figure 3 displays projected damaged areas of the laminates at different depths below the impacted surface. Damages are displayed as bright areas on a dark background. It was found that the damage occurred unevenly across the thickness. Interplay delaminations were found in three zones (Figure 3): upper, middle and lower, which corresponded to a depth range of 0.4 ÷ 0.8 mm, 2.0 ÷ 2.8 mm and 3.3 ÷ 4.4 mm (left, central, and right column, respectively) with some variations. For each type of laminate, damages in the upper zone close to the impacted surface are characterized by a minimal size of delaminations that appear from the third up to the seventh interplay (left column). Damages in the middle of the laminates have a maximal square and most of them are located around the central 90–90° interface between 17th and 22nd plies. Delaminations in the lower part of the laminates located from the 27th to 37th interfaces decrease in size as depth increases (right column).

**Figure 4:**
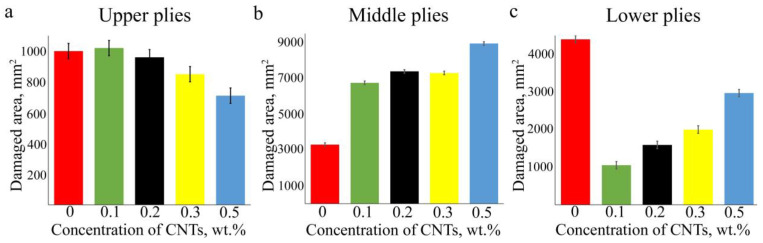
Diagrams showing the distribution of damage zones in laminates depending on CNT concentration. The addition of CNTs reduces the damage in the top (**a**) and bottom (**c**) plies, while increasing it in the middle (**b**) plies. The spread of values relates to the method used for calculating the damaged area in C-scans.

Figure 4 shows the histograms of the correlation between the damaged areas in three regions and CNT concentration. Based on ultrasound data, it can be concluded that the addition of CNTs to the matrix has two distinct effects. First, it reduces the damaged areas in the upper and lower parts of the laminates. Second, it increases the central damaged area. The central damage of the laminates with CNTs extends to the edges, which can affect the spread of delaminations, while the laminate without filler seals delaminations that can affect the size of the damage located in the lower part of the laminate. The total area of delamination observed in the volume of laminates increases with increasing concentration of CNTs; however, this does not mean a decrease in the residual strength of the laminates, which depends on the direction of damaged layers and broken fibers, as well as on the location of delamination in the thickness of the laminate.

### 3.4. Layer-by-Layer Imaging of Damage of the Laminates

**Figure 5:**
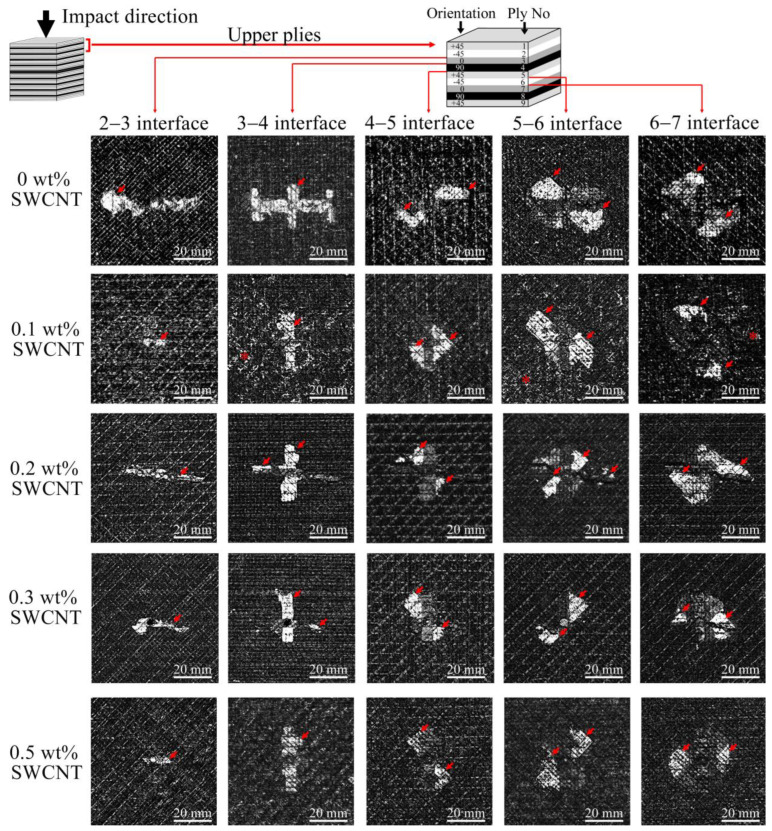
Layer-by-layer C-scans of damages in upper plies of impacted laminates with various CNT concentrations (from up to down). The size of damage decreases with increasing concentration of CNTs. Arrows depict the interlayer delaminations, stars are CNT conglomerates clearly observed in the laminate with 0.1 wt% of CNTs. The shape of the delaminations is determined by the orientation of the fibers in adjacent plies. Delaminations appeared in each interlayer from the second to the seventh.

Layer-by-layer ultrasonic imaging of the damage depicts the delamination that appeared simultaneously in each interface of the damage zones. Figure 5 shows the sequences of damage propagation in the upper plies of the laminates. The interlayer delaminations are displayed as bright areas against the dark background that depicted fiber orientations in adjacent plies. Damage occurs at the 2–3 interface and below; delaminations spread along the fiber orientation of neighboring plies of the interfaces. Usually, the laminates have double triangle-shaped delaminations symmetric to the impact point. The exception is the 0–90° boundary, where the front of the delamination development has a rectangular shape. The development of delaminations from the impacted surface comes about due to the rotation of damaged areas, with jumps from one interface to another. It was also found that the laminates with 0.1 and 0.5 wt% of CNTs had a conglomeration of fillers that can be seen in the C-scans as bright small-scaled elements (marked with a star in Figure 5). Therefore, the ultrasound contrast decreased close to the middle zone of the laminates (Figure 3). Nevertheless, the inhomogeneous distribution of CNTs did not affect the impact resistance. The size of the delaminations in the upper plies decreased with increasing CNT concentration.

**Figure 6:**
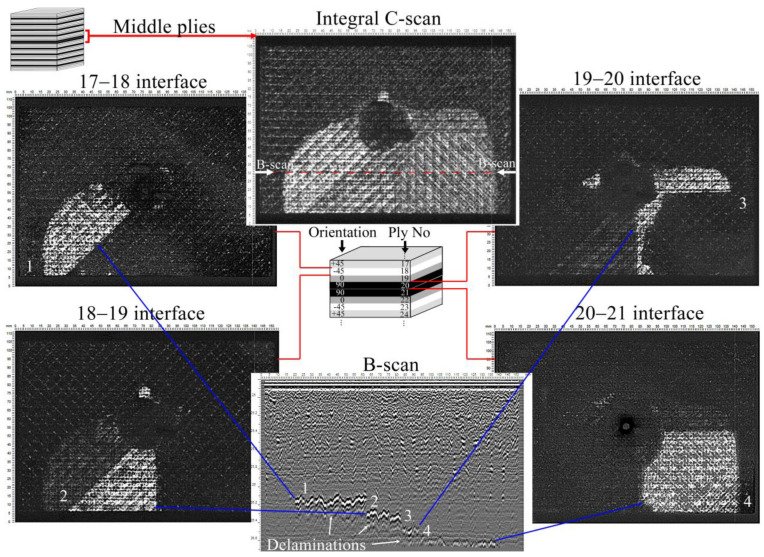
Layer-by-layer C-scans of damages in the middle plies of the laminate with 0.3 wt% of CNTs. Damages appeared step by step in four interfaces that were seen in the B-scan. The position of the B-scan is marked in the integral C-scan showing the total amount of damage in the middle zone. The shape of the delaminations is determined by the orientation of adjacent plies. The orientation and numbering of the plies are shown in the scheme. C-scans show a damage distribution typical for all tested laminates.

On the other hand, the damage in the middle plies had the largest area (Figure 4), and the total area increased with the increase of CNT concentration. These damages were also distributed across multiple interfaces. Figure 6 shows the damage distribution in the middle of the laminate with 0.3 wt% of CNT. A similar damage propagation mechanism was observed in the other laminates. The mechanism was that the damage appeared step by step (B-scan, Figure 6) at the four interfaces, and the largest delamination occurred at the 20–21 interface (90–90° layers). The smallest delamination was observed between the 0° and 90° layers (19–20 interface), which can be explained by the largest angular differences in fiber orientations. The distribution of damage across the interfaces ensured the integrity of the laminates and their residual strength.

**Figure 7:**
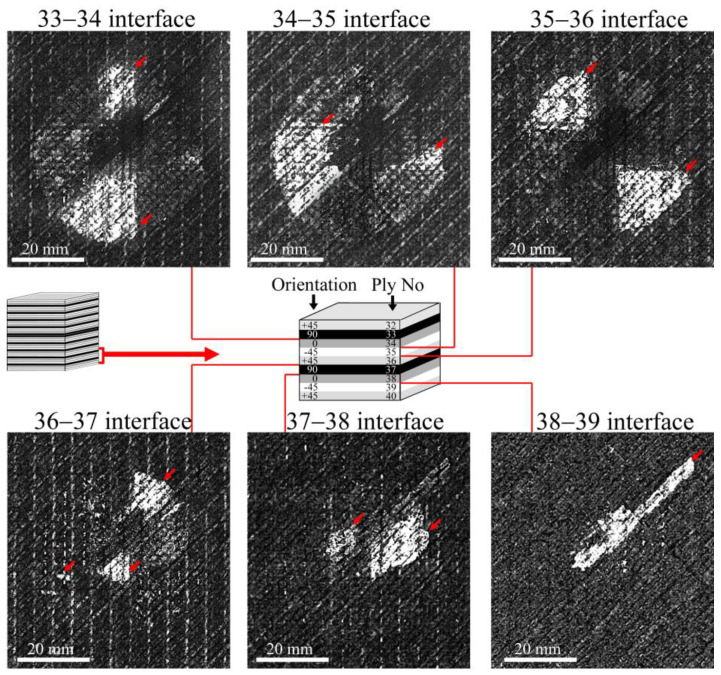
Layer-by-layer C-scans of damages in the lower plies of the laminate with 0.2 wt% of CNTs. Maximal delaminations formed close to the 30 ply and decreased towards the back surface of the laminate. The shape of the delaminations is determined by the orientation of adjacent plies. The orientation and numbering of the plies are shown in the scheme. C-scans show a damage distribution typical for all tested laminates with CNT filler. Arrows are the interlayer delaminations.

The damages in the lower plies of the laminates appeared from the 33rd ply up to the back surface of the laminates. Figure 7 shows the damage occurrence in the laminate with 0.2 wt% of CNTs. The delaminations have the shape of a symmetrical triangle with the maximum area at the interfaces from the 33rd to the 36th layers; then the size of the damage decreases, with delaminations observed up to the 39th ply. A similar damage propagation was observed in the other laminates with the maximal delaminations close to the 30th ply. The shape and front of the delaminations are determined by the orientations of the fiber in neighboring plies.

## 4. Discussion

The main action of nanofillers in the CFRP laminates is to inhibit crack growth between the fibers and the matrix, which leads to effective energy absorption. It is known that improvements in mechanical properties and efficient load transfer from the fibers to the matrix depend on the interfacial adhesion between CFRP components [12,46,47,48]. The inclusion of nanoparticles causes a change in interlayer interaction to intralayer, which results in an increase in the resistance of the matrix to cracking. In this work, it was found that the efficiency in facilitating the impact energy transfer between the fiber and matrix increases with increasing CNT concentration. The area of damage in the upper part of the laminates decreased (Figure 3). CNTs embedded between carbon fibers (Figure 1) can increase the interactions between plies along the direction of impact (vertically) and delay interlayer delaminations in the upper zone of laminates. The increased interaction between plies in laminates with CNT results in the transfer of impact energy from the upper plies to the middle region, where maximum stress was caused by the accumulation of energy; its abrupt release led to extended interlayer delaminations.

Based on the obtained ultrasonic data, the scheme of delamination location can be summarized as follows. The damage appeared from the second layer under the impactor. The size of the delamination increased from interface to interface up to the 8th ply of the laminates, after which the damage was not visible until the 17th ply, where the maximum delaminations formed step by step on the four lower interfaces. Further damage was observed only under the 30th ply, where delaminations spread from ply to ply in decreasing size. In combination with the impact history, it can be summarized that the damage initiated at the impactor’s deflection, about 0.7 mm (Figure 2c), corresponded to the depth of damages in the upper plies seen in the C-scans. (Figure 3). Similar damage initiation energy in all laminates did not affect the occurrence or propagation of damage in the upper layers of the laminates. Moreover, slightly lower *E_ini_* values (Table 1) were found in laminates with 0.1 and 0.5 wt% of CNTs. This might be due to the cluster distribution of the filler (Figure 3) which could affect the redistribution of stress in the volume of the material. In addition, a correlation between the absorbed energy *E_ab_* (Figure 2b) and the integral area of damage was clearly traced, which had a dependence close to linear. An increase in the filler concentration led to an increase in the absorbed energy from 13.5 to 18.2 J. At the same time, the sum of damaged area observed in the upper, middle, and lower plies of the laminates with various CNT concentrations (Figure 4) successively increased from 8700 to 12,600 mm^2^. It should be noted that the damage size increased in the middle layers of the laminates with filler (Figure 4), thus it can be assumed that most of the absorbed energy was spent on the middle plies. The addition of CNTs into the polymer matrix significantly affected the damage in the upper and lower plies of laminates, the size of which was two times smaller compared to damage in a laminate without CNTs (Figure 4). In addition, damage in laminates without CNTs had a larger spread of damaged interfaces in the region of the lower plies. Typically, these outer upper and lower areas of laminates influenced the residual strength of the material [49].

Thus, all parameters of damage in the volume of laminates, namely: the size of delaminations, their location and depth, the sequence of damage propagation, the orientation of the fibers of the damage layers, etc., can affect the future stability of laminates, which requires additional experiments to determine the correlation between the distribution of damage in the volume of laminates with CNTs and their residual compressive strength after impact.

## 5. Conclusions

In the work, the low-velocity impact damage appearance and propagation in the biaxial fabric laminates with various concentrations of CNTs in a polymer matrix was investigated. It was found that CNTs clustered together and formed a continuous transverse network between carbo fibers, and the size of the clusters increased with increasing CNT concentrations. Visualization of the damages in the volume microstructure was carried out using a high-frequency ultrasound imaging method, which allowed layer-by-layer localization and observation of the distribution of interlayer delaminations. Based on the data obtained, the following can be concluded:

− The addition of CNTs to the matrix reduces the damaged areas in the outer plies of the laminates; the size of damages was two times smaller compared to damage in a laminate without CNTs.
− Increasing the concentration of CNTs increases interlayer interaction and impact energy transfer through the thickness, which affects the energy absorption in laminates and increases damage in the middle region.
− CNT conglomeration affects the stress distribution in laminates, which reduces the threshold load for delamination.

We suppose that the low damages in outer upper and lower areas of laminates will influence the residual strength of the material both under static and cyclic loads.

In the future, it seems attractive to continue the work on identifying impact damage in CFRP with nano-sized filler and determining the dependence of localization and size of damages on the residual strength of laminates.

## Figures and Tables

**Table 1:** Impact data on delamination threshold load and peak force of the specimens.

Specimen	0 wt%	0.1 wt%	0.2 wt%	0.3 wt%	0.5 wt%
Threshold load *F_d_*, kN	4.25	3.90	4.44	4.55	3.40
Peak force *F_max_*, kN	11.80	11.68	11.84	10.80	11.42
Initiation Energy *E_ini_*, J	2.10	1.70	2.70	2.80	1.80
Energy to peak force *E_max_*, J	29.73	27.62	27.98	23.22	27.85
Absorbed Energy *E_ab_*, J	13.50	15.50	17.30	16.40	18.20

## Data Availability

Data is contained within the article.

## Abbreviations

The following abbreviations are used in this manuscript:

CFRP	Carbon fiber reinforced polymer
CNT	Carbon nanotube
